# Exchange Counterion in Polycationic Hydrogels: Tunability of Hydrophobicity, Water State, and Floating Capability for a Floating pH Device

**DOI:** 10.3390/gels7030109

**Published:** 2021-08-05

**Authors:** Martin Danko, Zuzana Kronekova, Igor Krupa, Jan Tkac, Peter Matúš, Peter Kasak

**Affiliations:** 1 Center for Advanced Materials, Qatar University, Doha P.O. Box 2713, Qatar; igor.krupa@qu.edu.qa; 2 Polymer Institute, Slovak Academy of Sciences, Dúbravská cesta 9, 845 41 Bratislava, Slovakia; zuzana.kronekova@savba.sk; 3 Institute of Chemistry, Slovak Academy of Sciences, Dúbravská cesta 9, 845 38 Bratislava, Slovakia; jan.tkac@savba.sk; 4 Institute of Laboratory Research on Geomaterials, Faculty of Natural Sciences, Comenius University in Bratislava, Mlynská dolina, Ilkovičova 6, 842 15 Bratislava, Slovakia; peter.matus@uniba.sk

**Keywords:** counterion exchange, hydrophobic hydrogel, water state, swelling, floating device

## Abstract

Smart gel materials are capable of controlling and switching swelling, water state, and wettability properties triggered by external stimuli. In this study, we fabricated a series of polyelectrolyte hydrogels bearing a 3-trimethylammoniumpropyl pendant to a methacrylamide-based backbone and examined the switchability with hydrophobic-like counteranions. The exchange between the initial chloride and camphor sulfate (CaS), dodecyl sulfate (DS), and perfluorooctanoate (PFO) counterions was investigated. The kinetics of the exchange showed that the fast exchange (within 4 h) of PFO allowed for a favorable coordination for ion pairing, resulting in a decrease in hydration. The reversibility of the exchange to the Cl^−^ ion was only enabled for the CaS ion due to its bulkiness, while the PFO and DS hydrogels were unable to exchange, even by using tetrabutylammonium chloride, which is a structurally similar reagent, due to aggregation or the coagulates in the collapsed state of the linear tails of the counterions. The hydrogels exhibited a modulable water state and water swelling. Moreover, the hydrogels containing DS and PFO, as counterions, showed surface hydrophobic (contact angle 90°) and high hydrophobic (110°) behavior, respectively. The Raman spectrometry fluorescence with a pyrene probe indicated an increase in strong hydrogen-bonded water molecules, water confinement, and hydrophobic domains in the PFO hydrogel. Moreover, the PFO-modified hydrogel demonstrated a free-floating ability on the water surface, with a strong water repellency, showing that it has the potential to be applied in a floating pH detection device to distinguish between volatile and nonvolatile bases in a controlled manner.

## 1. Introduction

Smart gel materials have been paid a lot of attention by researchers in various fields, such as tissue bioengineering, drug delivery, (bio)sensoring, display devices, and advanced energy applications [1,2,3,4,5]. This attention has resulted mainly from the capability of these materials to control and switch swelling properties, triggered by external stimuli [1]. Primarily gel swelling properties can be modulated by the combination of polymer chains with a swelled medium, such as water or organic solvent, and/or by the crosslinking the density of a network [6]. A part of controlled swelling, water structure plays a major role in the field of medical device coatings and bio-electronics. In the water structure, water molecules are attracted to the polymer chain, as a non-freezable bound, allowing freezable water to be influenced by the monomer, architecture, modification, or crosslinking [7]. Thus, the design and structure of the polymeric network are subsequently tailored to external stimuli triggering and particular applications [8]. The type of polymer chain usually dictates the response to external stimuli, such thermoresponsibility (mainly poly(*N*-isopropylacrylamide) [9,10] and zwitterionic-based material [11] due to the lower and upper critical solution temperature, respectively), light responsibility (azo-, spiro-, and 2-nitrophenyl-based materials) [12], electrical potential (charged or redox-based material [13,14]), pH (acidic and/or base-containing materials) [15], etc.

Another approach to dynamically and controllable adjust properties is counterion exchange. Materials with counterion exchange predominantly possess a quaternary ammonium moiety as a stable cationic group [16]. Apart from the tuning of the swelling behavior, counteranions are responsible for changes in the electrical conductivity, water state, and wettability. It was observed that in self-assembly monolayers [17,18], the layer-by-layer (LBL) design [19,20,21,22,23], conductive polymeric films [24], imidazolium-based anchored carbon nanotubes [25], and quarternized amine polymer brushes [26,27,28,29], an enormous change in wettability between counterions was observed. The exchange between Cl^−^ to the perfluorooctanoate ion is the most dramatic; it leads to a controlled exchange between superhydrophilic and superhydrophobic behavior, respectively.

Polyelectrolyte gels possess a charged polyionic entity—either a polycationic or polyanionic polymer backbone—and can be a suitable candidate for counterion exchange-based smart materials [30]. Their swelling properties are a combination of electrostatic interaction charges on the polymer chains and counterion and the osmotic difference in the interior and exterior of the gelled material. It was observed that counterions dramatically influence swelling, water, and conductivity [31]. Most of these gels attract water and have largely non-freezable and freezable bond water and attract water with a very low water contact angle. Besides, PFO, as a counterion in polyelectrolyte-polydimethylsiloxane-based hydrogel, was the best counterion candidate in terms of the anti-icing properties of a hydrogel-based coating [32,33]. It should be mentioned that a high water contact angle in a polyelectrolyte hydrogel is rather rare. Water repellency can be modulated by copolymerization or counterions. A polyelectrolyte gel with a specific absorption volatile organic compound using large hydrophilic counterions was proposed, which led to the formation of materials capable of absorbing highly volatile organic compounds [34,35,36,37]. In this case, quaternary ammonium monomer derivatives were usually copolymerized with a monomer with a long aliphatic tail. Another fabricated hydrogel with a specific absorption is based on the formation of a semipermeable skin layer in the presence of a good solvent in hydrogel rapid phase separation, and the asymmetric diffusion of water molecules into the gel was driven by the high osmotic pressure of the organic solvent–water mixing [38]. It is noteworthy that an approach with a liquid marble-like strategy led to a hydrogel with hydrophobic properties. This approach required the modification of the outer part using perfluorinated-modified diatoms, keeping the inner part intact [39].

Thus, in this study, we synthetized a series of polyelectrolyte hydrogels possessing a monomer with a trimethylammonium pendant. The fabrication of hydrogels was varied by the amount of crosslinker and the addition of a charge-free “passive monomer”, 2-hydroxyethylacrylamide. Subsequently, counterion exchanges with different anions, such as camphor sulfate (CaS), dodecyl sulfate (DS), and perfluorooctanoate (PFO) ions, were studied. The water state, surface water contact angle, and water swelling were studied, showing the hydrogels’ controllable tunability of such parameters after counterion exchange. Moreover, the PFO-based hydrogel revealed a strong hydrogel bonding of water molecules, hydrophobic domains, and free-floating on the water surface, which are attractive features for a floating pH detection device.

## 2. Results and Discussion

### 2.1. Hydrogel Formation and Its Characterization

**Scheme 1:**
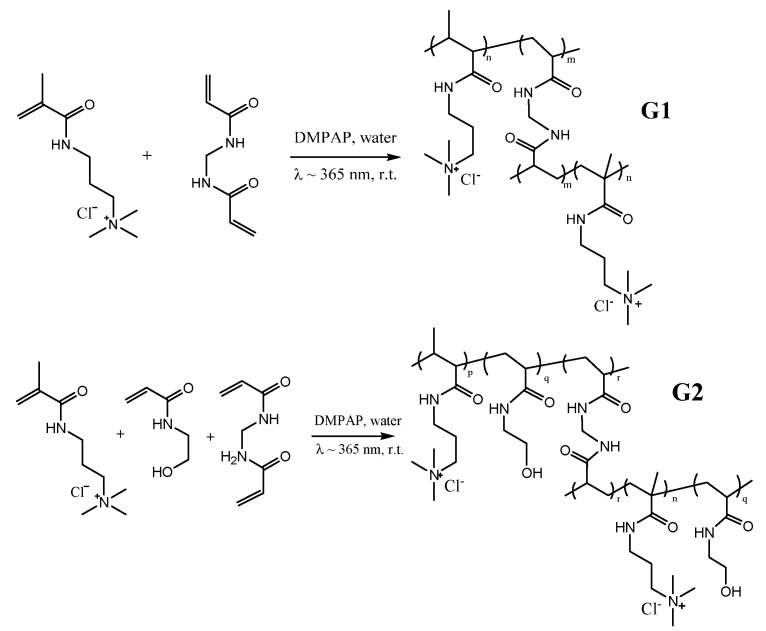
Reaction schemes of the prepared G1 and G2 gel series.

Polycationic gels were prepared by the free radical crosslinking polymerization of a 3-(methacryloylamino)propyl]trimethylammonium chloride (MTAC) monomer (gel series G1) or a mixture of cationic MTAC and a neutral *N*-(2-hydroxyethyl) acrylamide (HEAA) monomer (gel series G2) crosslinked by *N*,*N’*-methylenebis(acrylamnide) (MBA) and photoinitiated by 2,2′-dimethoxy-2-phenylacetophenone (DMPA), as is schematically described in Scheme 1. The composition of monomers and the amount of crosslinker in the final hydrogels are listed in Table 1. We fabricated the first hydrogel from either a quaternary ammonium (meth)acrylamide-based monomer MTAC, with a concentration of the monomer of 2 M in the feed and various concentrations of the MBA crosslinker, starting from 1–5%, as depicted in Table 1, entry 1–4. The second type of hydrogel, shown in Table 1, entry 5–8, was fabricated in an equimolar ratio of MTAC and HEAA, with an overall concentration of the monomer of 2 M and different MBA crosslinker concentrations in the feed. The monomer, HEAA, was chosen, since it is highly water-soluble, it is not expected to change, and it has the same functionality in the polymeric methacrylamide backbone. Moreover, polymer materials based on HEAA are highly biocompatible and prevent biofouling [40,41], which make them attractive for biomedical and bioengineering applications. As the prepared gels were immersed, the physiological solution was equilibrated in a repeated manner to remove unreacted monomers and low-molecular-weight species. A different extent of swelling was observed as a function of the crosslinking density (Table 1). In the equilibrium state, the gels swelled suddenly. In this context, a higher swelling has been observed for MTAC-based polycationic gels, G1. In both cases, the swelling degree was consistently lower for the denser network with an increasing crosslinker content. Due to the polyionic nature of the gels, the gels exhibit a standard polyelectrolyte effect, which was responsible for its higher swelling in pure water.

The FTIR analysis of xerogels G1 and G2 after a freeze-drying process, described in Figure S1 (in Supplementary Materials), showed a C=O stretching vibration at 1630 cm^−1^ and N–H bending vibration at 1530 cm^−1^, which were attributed to the amide group and bending C-H vibration at 1535 and 1479 cm^−1^, respectively, as characteristic vibrations of the alkyl-methacrylamide-based polymer backbone. Moreover, the G1 hydrogel showed an absorption peak at 1201 cm^−1^, which corresponds to the N-C stretching vibration of quaternary ammonium pendant moieties. G2 gels also exhibit an absorption band at 1063 cm^−1^ ascribed to the C-OH bending vibration, which is absent for the G1 gels, and a dramatic decrease in the stretching vibration at ~1200 cm^−1^, which is due to the N-C stretching vibration from the ammonium group. A strong hydrogen bonding from the N-H and –OH groups are supported by the absorption peak observed at 3300 cm^−1^ in all hydrogels.

To obtain data about the mechanical properties and compare each type of hydrogel, compression tests were conducted in the swollen equilibrated hydrogel samples, and the results are presented in Table 1. Both the toughness and elongation, as the break compression testing parameters, follow the trend of the crosslinker content and thus the crosslinking density. The parameter, σ, increases with the increase in the crosslink density and varied from 100 kPa up to 500 kPa for G1-1-Cl and G1-5-Cl, respectively. In the case of G2, the range is smaller and varied from 200 kPa up to 450 kPa for 1 and 5 mol% of MBA, respectively. These values are approx. 10 times higher than those obtained for methacrylate sulfobetaine-based polyzwitterionic hydrogels, where 1 mol% of the MBA, as a crosslinker, was used [42], or the sulfobetaine double networking system [43] and hydrogels with the sulfobetaine crosslinker [44]. Hydrogels with between 2 and 5 mol% of MBA exhibit a relatively smaller increase in toughness, which indicates a sufficient crosslinking in samples. However, the elasticity progressively decreases as the crosslinker content increases. This is attributed to the low fraction energy due to the increase in the chemical crosslinking points [45]. Gels with 5 mol% of MBA lost their elasticity and became fragile. On the other hand, the 40–50% elasticity of softer G1-1-Cl and G2-1-Cl is typical for soft hydrogels. The G2 gels, where the cationic 3-trimethylammonium moiety in the monomer randomly copolymerized, with no charged neutral 2-hydroxyethyl groups, show a comparable toughness and slightly higher elasticity than the G1 gels.

### 2.2. Counterion Exchange Study

**Figure 1:**
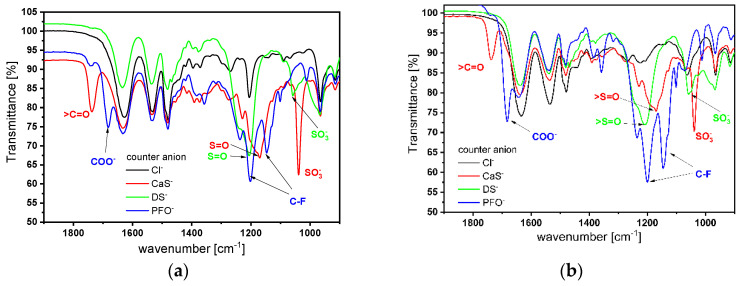

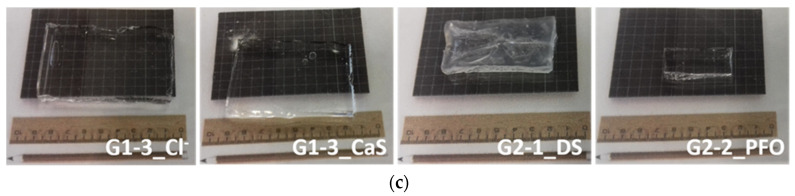
(**a**) FTIR spectra of the hydrogels from (**a**) the G1 series and (**b**) the G2 series, before (with the Cl counterion (black line)) and after the anion exchange equilibrium with CaS (red line), DS (green line) and PFO (blue line); (**c**) representative images of the hydrogels, before and after the exchange of the particular anion.

Previously, we (among others) showed that simple quaternary ammonium-based materials can simply reversibly change from a superhydrophilic to a superhydrophobic surface by counter ion exchange [17,22,25,26]. The simple exchangeability of ions led us to create hydrogels with hydrophobic properties. Due to this fact we scrutinized the commercially available hydrophobic counter anions. Three different counterions for anion exchange in cationic methacrylamide-based hydrogels were selected: dodecyl sulfate (DS) with linear alkyl chain, perfluorooctanoate (PFO), as a highly hydrophobic fluorinated example, and camphor sulfate (CaS), as a bulky and branched alkyl moiety, all with a similar number of carbons. Equilibrated and washed gels were immersed in a solution of various hydrophilic anions to allow for a spontaneous counter anion Cl^−^ exchange. Moreover, the possible rest of the unexchanged polymer pendant groups (3-trimethylammonium-propyl chloride) in the G1 series and 2-hydroxyethyl in the G2 series can serve as a hydrophilic phase to ensure a certain water retention in the gel. This is important for various applications, where both hydrophobic and hydrophilic moieties are required, such as for bio-applications involving hydrophobic drug delivery or for applications where so-called polysoap, which is similar to surfactant in certain properties [46], is needed. For screening how the anion exchange will affect the hydrogel samples, all gel formulations were immersed in a DS anion solution. Some pictures of the as-prepared gels, after washing and after anion exchange, are listed in Figure S2 (in Supplementary Materials). A dimension decrease was observed in all samples. The shape change, accompanied by an extensive loss of transparency, was observed in the G1-1-DS sample. On the other hand, the sample, G2-1-DS, with the same crosslinking density, has at least been affected by the anion exchange. Mechanically tough samples with a higher crosslinking density exhibit a loss of shape stability, with cracks and peeled parts in both series. From the abovementioned mechanical instability of some of the gel formulations, samples G1-2 and G1-3 from the G1 series and samples G2-1 and G2-2 from the G2 series were selected for the next experiments involving anion exchange. Anion exchange causes hydrogel dimension shrinkage, where hydrophobic anions expel water in the case of DS and PFO anions (Figure 1c). We assume that during the exchange, a hydrogel is formed, with a strong ion pairing between the quaternary ammonium and DS and CaS and PFO negative ions. This pairing led to a strong coordination and decrease in hydration energy, also leading to changes in the swelling of the gels. The changes in water swelling are summarized in Table 2. The amount of released water, W_R_, was calculated based on the theoretical anion uptake, where 50% and 100% of the Cl^−^ anions were exchanged in the G1 and G2 series, respectively (the same amount in both series). The range of water loss was 80–95% in comparison with the water content, before the anion exchange. Slightly higher water losses for the less dense G1-2 for both DS and PFO were observed. In the G2 series, no difference between anions or crosslink density was visible. Interestingly, in the case of CaS anion, the gels increased their weight and swelled by 10%. This is probably due to the bulky structure of the camphor-based moiety, with a reduced ability to become entangled with the hydrophobic part of the main polymer chain. On the other hand, there is a difference in behavior in comparison with the linear long alkyl chains of DS and PFO. Thus, no water is displaced from the hydrogel by CaS, while the entanglement of the linear alkyl moieties of the counterions with the polymer backbone in the case of DS and PFO led to a water release after the anion exchange.

The anion exchange was followed by FTIR spectroscopy. New absorption bands appeared for specific functional groups of hydrophobic anions after the Cl^−^ exchange (Figure 1a,b). A C=O stretching vibration at 1738 cm^−1^, S=O stretching at 1170 cm^−1^, and sulfate bending at 1039 cm^−1^ represented the presence of the CaS anion. The absorption bands at 1215 cm^−1^, with a shoulder at 1240 cm^−1^ and 1058 cm^−1^, are typical for the S=O stretching and SO_3_^−^ of DS. The strong carbon-fluor bending vibrations at 1202 and 1147 cm^−1^, with the C=O stretching vibration of the carboxylate anion at 1683 cm^−1^, are attributed to the PFO anion.

**Figure 2:**
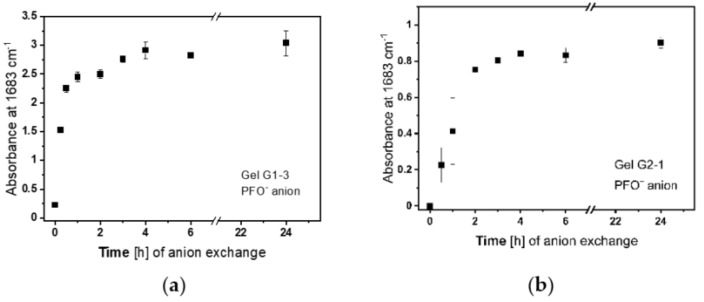
Kinetics of the PFO anion exchange in (**a**) the G1 and (**b**) G2 hydrogel series, monitored by FTIR at the 1683 cm^−1^ absorption band, which is typical for the PFO anion. c(PFO) = 0.05 and 0.034 mol L^−1^ for G1-3 and G2-1, respectively.

The kinetics of the anion exchange followed for PFO showed a rather fast exchange, achieving equilibrium after approx. 4 h for both types of gels (Figure 2). Here, a slower absorption band, increasing with the PFO anion, was observed for the G2 series, where the cations in the polymer backbone were substituted by charged-neutral 2-hydroxyethyl pendants, as in the case of the cation charge on every monomer of the polymer backbone in the G1 series. We assume that the exchange of PFO ions is instant and favorable due to the strong ion pairing coordination between the quaternary ammonium and PFO. It should be noted that the LBL (in seconds) [22] and self-assembly monolayer (in a dozen seconds) [17] exchange were much faster; however, in hydrogel slabs, the diffusion of PFO and Cl ions needs to be considered. In 500 µm of thick gel, Cl^−^ and PFO ion diffusion is considered to take place within a dozen mins [47], with a further subsequent relaxation of the polymer network. The kinetics of the exchange of DS were even slower and reached a plateau in 6 h, which can be attributed to the bulkier organo-sulfate ions in the diffusion process (Figure S3 in Supplementary Materials). 

The quantification of the anion exchange was followed only for the G1-3-PFO and G2-1-PFO gel samples by XPS analysis. The PFO anion has characteristic F atoms, which can be compared with the cationic N^+^. For the concentration of PFO/water solution used (0.05 mol L^−1^), the atomic% ratio of F/N^+^ was ~48% and ~95% for G1-3-PFO and G2-1-PFO, respectively, of the theoretical 15/1 ratio, assuming a full anion exchange. Thus, half of the counter anions remained unexchanged in G1-3-PFO, while almost a full exchange was achieved in G2-1-PFO. Because the G2 series contains 1/1 cationic MTAC and neutral HEAA comonomers, both gel types (G1 and G2) are comparable from the point of view of the PFO quantity.

**Figure 3:**
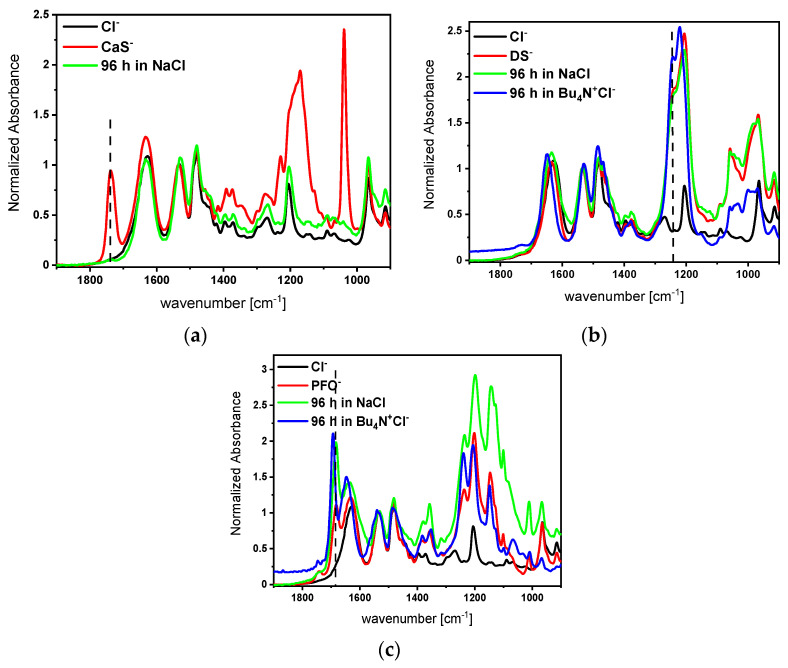
FTIR spectra of the samples attempting a reversible exchange to G1-3-Cl from (**a**) G1-3-CaS, (**b**) G1-3-DS, and (**c**) G1-3-PFO immersed in 3.5 wt% NaCl or Bu_4_N^+^Cl^−^ solutions. The line indicates the specific absorption band. The spectra were normalized on a polymer gel matrix absorption band at 1535 cm^−1^.

With the aim of obtaining gels with reversible counterion exchange properties, the samples were washed in 3.5 wt% NaCl or an organic salt tetrabutylammonium chloride (Bu_4_N^+^Cl^−^) solution to exchange the counteranion back to Cl^−^. It was observed that the CaS anion was exchanged quantitatively after 96 h by immersion in NaCl and Bu_4_N^+^Cl^−^, as revealed by the absence of the C=O absorption band at 1738 cm^−1^ in the FTIR spectra (Figure 3a). However, anions with long linear alkyl DS and perfluoroalkyl PFO chains were not removed from the gels, and a negligible exchange was observed at the same concentration after extraction (Figure 3b,c). It is known that PFO and DS anions coordinate with quaternary ammonium due to their hydrophobic-like character, which decreases the hydration energy [48]. Thus, we chose a structurally similar ammonium group, with even more electro-donating substituent Bu_4_N^+^ groups, as the cation in the exchange. We expected a favorable coordination, with a strong pairing to facilitate a reversible exchange. However, the change of inorganic salt for such an exchange reagent (Bu_4_N^+^Cl^−^) also did not lead to a successful chloride anion exchange. Considering the comparable strength of the ionic interaction of the sulfate anion of CaS and DS, the reversibility of the anion exchange of CaS^−^ to Cl^−^ could be attributed to the bulky and nonflexible nature of the camphor moiety, with a limited possibility for entanglement and being bundled within the polymer network, as discussed previously.

We hypothesize that the long perfluorinated PFO^−^ or aliphatic DS^−^ pendant form aggregates and/or coagulates, resulting from favorable van der Waals interactions between perfluoroalkyl or alkyl tails, respectively [49]. The resulting aggregates and/or coagulates contain such bundled tails in a collapsed state in the 3D network of the hydrogel, which hampers the exchange event with Bu_4_N^+^Cl^−^. Moreover, the presence of exchange ions might lead to a “salting out effect”, which restrains the anion exchange.

### 2.3. Water State in the Hydrogels

The water quantity and structure in the hydrogels are important from the point of view of further utilization, particularly in bioengineering and medical areas, where water states can influence the performance of cells and proteins and particular applications. We assume that the water in the swollen-hydrogel network can be defined as freezing free water (*W_ff_*—water molecules not involved in the interaction with the polymer chains), freezable bound water (*W_fb_*—water molecules with a weak interaction with polymer chains), and non-freezable bound water (*W_nf_*—water molecules with a strong interaction with polymer chains). In the present work, where hydrogels exchange anions, the water state characterization is an important feature of the gels. Both types of freezable water (*W_tf_*)—freezable bound and free—exhibit an exothermic phase transition below −10 °C (freezing) or endothermic phase transition between −5 and 5 °C (melting) in the DSC analysis and can be calculated according to Equation (1):

(1) $$W_{tf}=W_{ff}+W_{fb}=\frac{\Delta H}{H_{w}}\times 100$$

where ∆*H* is the melting enthalpy of the water in the hydrogel, which is determined by the integration of endotherm during the heating of the sample from DSC (Figure S6 in Supplementary Materials), and *H_W_* is the melting enthalpy of the bulk water, which equals to 333 J·g^−1^. The total or equilibrium water content (EWC) can be determined by freeze-drying as a difference between the weight of the water-swollen and totally dry gel. The difference between these two values determines the non-freezable water (Equation (2)), which does not exhibit a detectable exo/endo-phase transition over the temperature range −40 ± 20 °C.

(2) $$W_{nf\;}=EWC-W_{tf}$$

The contents of EWC, total freezing (*W_tf_*) water, and non-freezable water are listed in Table 3.

The EWC for all the equilibrated hydrogels with a Cl^−^ counterion lie, in both series, in the range of 84 to 96% for the highest and lowest crosslinker content, respectively, showing a large amount of water content. Increasing the cross-linking density further constrains the polymer gel and thus decreases the EWC values, as is progressively visible in Table 3. The rather high content of non-freezable water, which is highly associated with the polymer backbone interactions between 18% and 25%, can be attributed mainly to the (meth)acrylamide-based structural character of the used monomers capable of hydrogen bonding, the charged trialkylamino group, and the hydroxyethyl pendants. Comparable values of *W_nf_* ~20% were shown for the neat poly(methacrylate-sulfobetaine) polyzwitterion hydrogel [42]. Additionally, the water type is affected by the crosslinking density, since the values of freezing water *W_tf_* decreased gradually from 75% to 59% for G1-1-Cl and G1-5-Cl, respectively, and from 80% to 62% for G2-1-Cl and G2-5-Cl, respectively. Conversely, the non-freezable *W_nf_* water content remains rather independent of the crosslinker content in both the examined gel series, with values in the range of 15% to 25%, without the succession in growth with the crosslinker increasing. A dramatic difference was observed after the counter anion exchange. The electrostatic interaction of hydrophobic DS and PFO anions with the polymer network led to a water release from the gel structure, and the samples shrunk, as mentioned earlier. The EWC values decreased to 25–35% for G1-PFO/DS and to 45–70% for G2-PFO/DS. In the case of the G1 series, this water release was accompanied mainly by the release of freezable water molecules, where the *W_tf_* dropped to 18–25% for DS and to 12–15% for PFO, while the non-freezable water content was kept at approx. 20%. This behavior shows that strongly bonded water molecules close to the polymer backbones represented by amides and alkylamino cation moieties remained confined. However, the lipophilic alkyls of the DS and PFO counterions mainly expelled free water from the exterior of the polymer network. Conversely, the gels, after the CaS anion exchange, did not exhibit such hydrophobic behavior, and the EWC, *W_tf_*, and *W_nf_* parameters were similar to the original Cl^−^ containing G1 or G2 gels. This was also in accordance with the swelling experiments described above, where no shrinkage of the gels after the CaS anion exchange was observed. In the additional anion exchange experiment, where G1-3-Cl samples were immersed in a double anion concentration, the freezable water content dropped below 15%, while the observed non-freezable water content was approx. 40%. This effect is less pronounced in the G2 series with the hydroxyethyl moiety in the polymer backbone. Here, the freezable water content is still higher than approx. 30–60%, while the non-freezable water slightly decreased below 20%. The observed water distribution suggests that in the case of the G2-DS/PFO series, with the hydroxyethyl “softening” moiety possessing a lower water affinity than that of the alkylamino-charged moiety, the water is more evenly distributed in the gel framework. However, both series exhibit the ability to retain water in the 3D hydrogel structure.

### 2.4. Wettability of the Hydrogels

**Figure 4:**
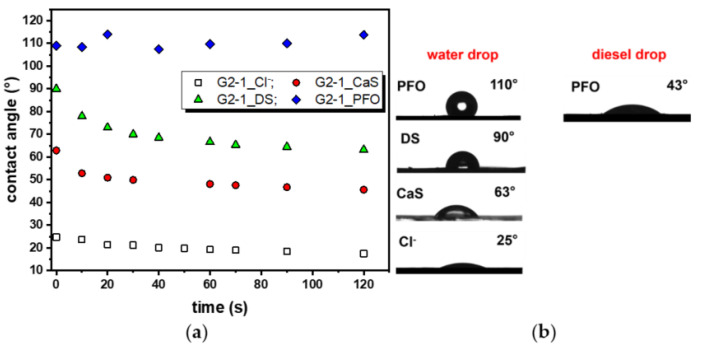
(**a**) Contact angles and time evolution of the contact angle on the surface of the G2-1 hydrogel type, before Cl (white square) and after the exchange with CaS (red bullet), DS (green triangle), and PFO (blue diamond) anions; (**b**) Photographs of 3 μL of water (left) or diesel (right) droplets, present on the surface at the beginning of the measurements.

The wettability characteristic of the surface of the hydrogels was investigated by contact angle measurements. The values of the contact angles and representative pictures of the water drop on the sample surfaces are summarized in Figure 4. These representative pictures were made for the G2-1 gels. It is necessary to mention that a similar wettability character in both the G1-3 and G2-1 gel series was observed. The contact angle of the water drop on the equilibrated hydrogel surface with the Cl^−^ counter anion was approx. 25°, with slow drop spilling at 17° over 2 min, showing the penetration of water into the hydrogel bulk. The hydrogel containing alkane-based anions led to an increase in the contact angle to values of 63° and 90° for the CaS and DS anion, respectively. In both cases, water drop spilling was observed over 2 min, and the values dropped down to approx. 20°, suggesting the possibility that the water penetrated into the bulk of the gel structure. The most hydrophobic surface was observed for gels with the PFO anion, where the water contact angles were up to 115°. The stability of the water drops, showing the robust and strong hydrophobic nature of these gel surfaces. Additionally, the lipophilic character of the PFO-based gels can be visible with a contact angle of 43° of the diesel drop, showing their wettability with such hydrophobic liquids.

### 2.5. Water Characteristics, Morphology, and Polarity Study

**Figure 5:**
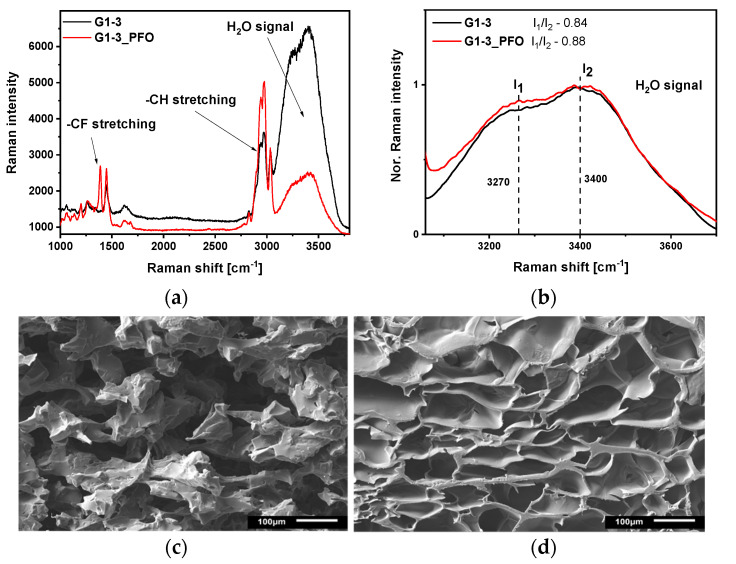

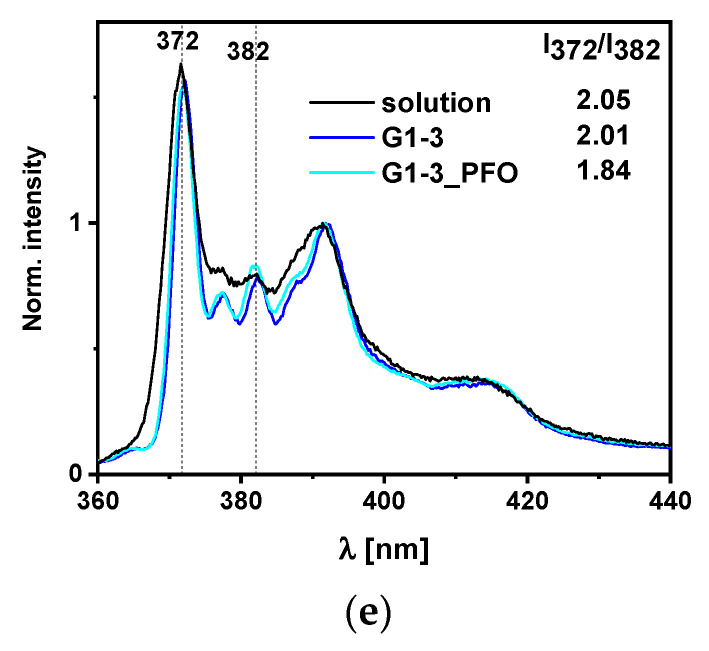
(**a**) Raman signals in the range of 1000–3800 cm^−1^ of the G1-3-Cl and G1-3-PFO gels at 100 µm below the gel surface; (**b**) Raman signal of the water stretching in the range of 3200–3800 cm^−1^ of the G1-3-Cl and G1-3-PFO hydrogels. The signal is normalized by weak water interaction vibrations at 3400 cm^−1^. Scanning electron microscopy (SEM) images of (**c**) the G1-3-Cl and (**d**) G1-3-PFO xerogels; (**e**) the fluorescence emission of pyrene (c = 1 × 10^−5^ M) in solution, the G1-3-Cl and G1-3-PFO gels, and an excitation of 344 nm in water/MeOH (1/1, *v/v*). The spectra were normalized at the 392 nm emission band.

Due to the uniqueness of the hydrogels, with a very hydrophobic character after the PFO exchange, we focus, furthermore, on the investigation of the water confinement, morphology, and polarity in the hydrogels after the exchange with the PFO anions (Figure 5). The water characteristics in the hydrogels were quantified using the Raman absorption band of water. A focused confocal layer at 100 µm from the top of the hydrogel, with a thickness of approx. 1 mm, exhibited water bimodal OH stretching, with characteristic Raman bands at approx. 3270 cm^−1^ (I_1_) and 3400 cm^−1^ (I_2_) for the G1-3-Cl hydrogel and G1-3-PFO, respectively, after the anion exchange (Figure 5b). The full Raman spectra of both gels are shown in Figure S4 (in Supplementary Materials). The main difference between the two spectra is the intensity of the water signals, which are in the range of 3100–3600 cm^−1^, in relation to the CH stretching bands of the alkyl pendant groups of the methacrylate polymer chains, which are at approx. 2970 cm^−1^ (Figure 5a). Considering the unchanged alkyl stretching before and after the anion exchange, the G1-3-Cl hydrogel exhibited a significantly higher intensity of the water band in comparison with the alkyl signal due to the higher water content. Conversely, this alkyl signal is stronger in the G1-3-PFO hydrophobic gel, where the water molecules were expelled from the gel after the anion exchange. The typical water signal in the range of 3100–3600 cm^−1^ was divided into two bands (Figure 5b). These bands are assigned to strongly (I_1_) and weakly (I_2_) hydrogen-bonded water molecules [50]. The observed I_1_/I_2_ ratio is higher for G1-3-PFO (0.88) than for hydrogel G1-3 (0.84). This is in accordance with our hypothesis, namely, that the free water molecules are in confinement, with a weak hydrogen bonding present in the G1-3 hydrogel that is partially expelled after the anion exchange in G1-3-PFO. Thus, the ratio of strongly hydrogen-bonded water molecules, which interact with the main polymer backbone remaining in the gel, is higher in the G1-3-PFO hydrogel [51]. As discussed, it is assumed that the hydrophobic counterions are in a collapsed state. Additionally, an SEM analysis was performed to study the porous structure of xerogels from the G1-3-CL and G1-3-PFO samples (Figure 5c,d). G1-3-Cl showed large symmetric pores in the range of 50 to 80 µm (Figure 5c), while the G1-3-PFO sample led to asymmetric pores and a dramatic reduction in the pore size to 15–50 µm due to less swelling (Figure 5d). Moreover, a thicker wall size of solid content was observed, with a heterogeneous pore size distribution from the interphase to the center of the hydrogel. A thicker wall, indicating a collapsed state, formed after the PFO addition, and smaller pores were observed in close proximity to the edge of the hydrogel, indicating a different distribution of the PFO ion, based on the diffusion of ions.

In addition, the polarity in the bulk of the gels was studied, using pyrene as a fluorescence probe. The pyrene probe diffused into the bulk interior of the gels, sensing its polarity through the polarity dependent intensity of the first vibronic band at 372 nm of the pyrene monomer emission. The ratio of the first and third (at 382 nm) I_372_/I_382_ emission is the so-called “pyrene scale” [52,53]. A more polar hydrophobic nature of the G1-3-PFO gel was expressed, with a lower I_372_/I_382_ ratio of 1.84, in comparison with the original G1-3-Cl gel or water/MeOH solution, where I_372_/I_382_ reached similar values of ~2 (Figure 5e).

Furthermore, the swelling properties in water-soluble protic polar (MeOH and EtOH) and less polar (*n*-BuOH and 1,7-heptane-diol) alcohols (Figure S5 in Supplementary Materials) were investigated. G1-3-Cl did not show an increase in swelling at all in the alcohols, compared to the water; it shrank in less polar BuOH and heptanediol to a greater extent than in EtOH. Interestingly, the copolymeric gel, G2-1-Cl, swelled to a greater extent in MeOH and especially in EtOH, but it shrank in less polar BuOH and 1,7-heptanediol and even to a slightly greater extent, compared with the shrinkage of the fully polycationic G1-3-Cl gel (Figure S5a in Supplementary Materials). The swelling in EtOH and partially in MeOH could be related with the hydroxyethyl pendant group of the comonomer, where the hydrogen interaction and solvent molecule dimension can play a role. After the anion exchange, the shrinkage of G1-3-PFO was observed in all solvents to a similar extent (Figure S5b in Supplementary Materials). In the G2-1-PFO gel, the difference between the significant swelling in MeOH and EtOH and significant shrinkage in BuOH and heptanediol was observed to be less significant.

### 2.6. Floating Capability of the PFO-Based Gel and Its Application as a Microdevice for pH Detection

**Figure 6:**
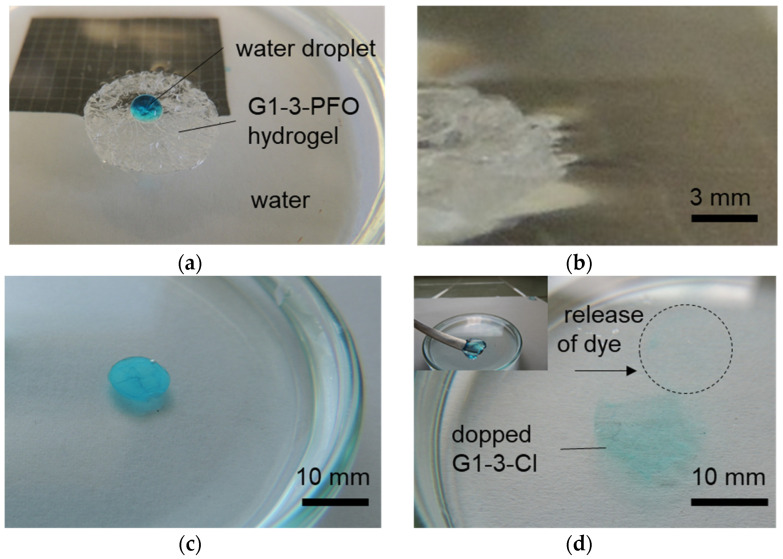
(**a**) Image of a water droplet (doped by methylene blue dye) on top of the G1-3-PFO hydrogel and floating in a water pool in a Petri dish; (**b**) image indicating the convex meniscus on top of the water, after the immersion of the G1-3-PFO hydrogel in the pool; (**c**) image of the G1-3-PFO hydrogel (doped with methylene blue dye) floating in the water pool in the Petri dish; and (**d**) image of the G1-3-Cl hydrogel (doped with methylene blue dye) sunk under water, with the inlet showing the dopped G1-3-Cl hydrogel before immersion.

Surface hydrophobicity can also be demonstrated on a floating slab of the G1-3-PFO hydrogel on a water tank, where the gel slab could easily float at the water/air interface, without sinking, as shown in Figure 6 and Video S1 in Supplementary Materials. The floating hydrogel remains on the water surface, even when the lab spoon is pressed against it, and no sinking is observed (Video S1, Supplementary Materials). Its hydrophobic character is also supported by the fact that, at the same time, the hydrophobic gel slab floating on the top of water shows a hydrophobic surface character, with a very high contact angle of the water droplet (visualized by doping with methylene blue dye) on the top of the slab (Figure 6a). Moreover, the repellency is revealed by the observation of the convex meniscus with entrapped air bubbles, which are formed in the water in proximity to the interphase between the water and air (Figure 6b). Additionally, no release of methylene blue dye from the floating doped G1-3-PFO hydrogel is observed (Figure 6c). Importantly and interestingly, the hydrogel G1-3-PFO shows a water-repelling ability, despite the water present in the bulk hydrogel body (please see the section in this paper concerning the water state). This suggests that besides the general water state inside the network (EWC for G2-1-PFO is 45%, as listed in Table 3), the hydrophobic counterion, along with the polymer frame hydrophobic layer with entrapped air, forms an interface, which allows the gel slab to float on the water. This floating character of the hydrogel, with a water repellency behavior, can be ascribed to a state that results in an initially preferred Cassie−Baxter state [54]. Contrary to the typical Wenzel state, the water from both the environment-hydrogel and water tank should diffuse and, together with dye, penetrate the hydrogel structure and water tank, which would result in an increase in the solid−liquid contact area [55]. It should be noted that the G1-3-Cl hydrogel sink, which is under the water due to the higher density of the poly(meth)acrylamide copolymer hydrogel (approx. 1.03 g cm^−3^), compared to the water in the pool and the methylene blue dye, was immediately diffused and released from the sunk hydrogel (Figure 6d). Previously, hydrophobic hydrogel required floating liquid marble technology [39]. Thus, according to our knowledge, this is the first report of a hydrophobic floating-type hydrogel based on a counterion exchange in the hydrogel.

**Figure 7:**
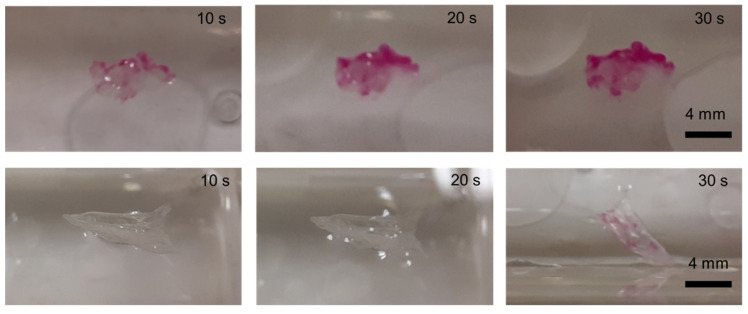
Time-lapse images indicating the color change of the hydrogel doped with phenolphtalein, as a floating pH meter for NH_3_ (**upper**) and NaOH (**bottom**) solutions.

Furthermore, due to the preference for the Cassie Baxter state at the beginning of floating, the hydrogel in the interphase doped with pH indicators (phenolphthalein) was used. The hydrogel pH floating device in contact with a basic solution containing volatile NH_3_ led to a gradual and progressive change immediately after the hydrogel immersion due to the evaporation of NH_3_ and subsequent penetration of the NH_3_ vapor into the floating pH device (Figure 7 upper). On the other hand, in the case of a nonvolatile base, such as NaOH, this did not result in an immediate change of pH in the floating hydrogel device, beginning at the edges of the hydrogel sample after 30 s of floating (Figure 7 bottom). Despite the broad availability of pH probes and sensing setups, devices, and materials, reports on floating devices to distinguish between volatile and nonvolatile bases in a controlled manner are rare.

## 3. Conclusions

In this study, we synthesized a set of polyelectrolyte hydrogels with a 3-trimethylammoniumpropylmethacrylamide monomer as the polycationic segment and chloride as the counterions. The switchability to camphor sulfate (CaS), dodecyl sulfate (DS), and perfluorooctanoate (PFO) counterions was studied, showing a favorable coordination for ion pairing, decrease in hydration, and non-reversible exchange character for PFO and DS. However, an increased swelling and reversibility in the case of the CaS counterion, due to its bulk and branched character, with a bicyclic structure, was observed. The hydrogels showed a modulable water state and water swelling, and the hydrogels containing DS and PFO as the counterions showed surface hydrophobic (water contact angle 90°) and high hydrophobic (water contact angle 110°) behavior, respectively. The investigation of the PFO-modified hydrogel revealed strong hydrogen-bonded water molecules, water confinement, an asymmetric pore distribution, and hydrophobic domains. Additionally, the PFO-modified hydrogel demonstrated a free-floating capability on the water surface, with strong repellency, showing a convex meniscus with an initially preferred Cassie state on the water-air interphase. To our knowledge, this is the first report on hydrophobic floating hydrogel based on the counterion exchange in the hydrogel. Moreover, the G1-3-PFO hydrogel was applied for a controlled floating pH detection device, distinguishing volatile and non-volatile bases in a timely manner. Previously, hydrophobic hydrogel required liquid marble technology [40]; thus, according to our knowledge, this is the first report on hydrophobic floating-type hydrogel based on the counterion exchange in the hydrogel.

It is noteworthy that the PFO anion is widely applied as a surfactant in the process of poly(tetrafluoroethylene) (Teflon) production [56,57]. Due to its low ecological degradability and abusive effect on human health [58,59], it is considered a contaminant in water [60], and the removal process is in high demand. The studied hydrogel shows a fast exchange of PFO ions in the hydrogel structure and using it to create a floating device might be attractive in relation to the removal of PFO as a pollutant. This study is ongoing in our labs.

## 4. Materials and Methods

### 4.1. Materials

A 3-(Methacryloylamino)propyl]trimethylammonium chloride water solution (monomer MTAC, 50/50, *v/v*, Merck KGaA, Darmstadt, Germany), *N*-(2-hydroxyethyl) acrylamide (monomer HEAA, 97%, Merck KGaA, Darmstadt, Germany), *N*,*N’*-methylenebis(acrylamide) (MBA, 99%, Merck KGaA, Darmstadt, Germany), and 2,2′-dimethoxy-2-phenylacetophenone (DMPA, 99%, Merck KGaA, Darmstadt, Germany) were used as received. Sodium perfluorooctanoate and sodium camphor sulfate were prepared by the dissolution of perfluorooctanoic acid (98%, Merck KGaA, Darmstadt, Germany) and (+)-camphor-10-sulphonic acid (99%, Sigma-Aldrich), respectively, in the presence of an equimolar amount of NaOH. Sodium dodecyl sulfate (Riedel de Häen) was used as received. Pyrene (Py, 99+% for fluorescence, Fw = 202.25, Sigma-Aldrich), methylene blue hydrate (95%, Merck KGaA, Darmstadt, Germany), and tetrabutylammonium chloride hydrate (Bu_4_N^+^Cl^−^, 98%, Merck KGaA, Darmstadt, Germany) were used as received. Deionized water (DI water) was obtained from a milliQ Millipore setup. Methanol (MeOH), ethanol (EtOH), *n*-butanol (*n*-BuOH), and 1,7-heptanediol (hept) (Merck KGaA, Darmstadt, Germany) were analytical grade solvents and were used as received.

### 4.2. Hydrogel Preparation

Hydrogels were prepared by the photoinitiated free radical polymerization of the cationic monomer, MTAC, or a mixture of cationic and neutral MTAC+HEAA in the presence of an MBA crosslinker. The component composition ratio in the feed and its appropriate amounts needed for a particular hydrogel are listed in Table 1. In a typical polymerization process, the monomer(s) was dissolved in DI water. An appropriate amount of MBA crosslinker pre-dissolved in 150 µL of ethanol was added to the monomer/water solution and mixed, until the MBA was dissolved. The DMPA photoinitiator (1 mg) was dissolved in 100 µL of EtOH and added to the monomer mixture, just before polymerization. The mixture was then poured into a glass mold made of two square glass plates sealed by a silicon tube. The thickness of the mold was 1 mm, set up by a distance glass plate placed behind the seal on both sides. The polymerization was performed under 365 nm of light irradiation in a UV chamber, Spectrolinker XL-1500 (Spectronics corporation), with an intensity of 1200 J/m^2^ for 90 min. After that, the hydrogels were carefully separated from the glass plates using a wet spatula and immersed in a 0.9 wt% NaCl/water solution.

### 4.3. Anion Exchange

Equilibrated gels were cut to dimensions of 1.0 × 1.0 cm using a razor blade and immersed in an anion/water solution for 24 h in order to exchange the Cl^−^ anion for the hydrophobic anion, camphor sulfate (CaS), dodecyl sulfate (DS), and perfluorooctanoate (PFO). The concentrations of the anions were calculated to the exchange whole amount of Cl^−^ anions in the G1 and G2 series gels. The monomer concentration varied in particular gels, based on their volume and degree of swelling after equilibration. In particular, the gel slabs were immersed in small Petri dishes with 2.5 mL of an anion/water solution, and the concentration of anions c = 0.034 mol L^−1^ for the G1-2-Cl and G2-1-Cl gels and c = 0.05 mol L^−1^ for the G1-3-Cl and G2-2-Cl gels. Two approaches to anion exchange were applied. In the first approach, the gels were immersed in the final anion concentration for 24 h. In the second approach, the gels were immersed gradually in 1/10 of the anion concentration, followed by immersion in 1/4, 1/2, and the final concentration, until the equilibrium was reached in a particular step (in general, at 4 h). After that, the gels were washed in deionized water to remove any non-specifically surface-adsorbed anions, prior to the subsequent characterization.

The degree of gel swelling (DSw) in a particular step was calculated as the ratio of the sample weight *m_Cl_^−^*, *m_w_*, and *m_A_^−^* equilibrated in 0.9 wt% NaCl, deionized water, and after the anion exchange, respectively. The original gel, after the preparation *m*_0_, was calculated according to Equations (3)–(5):

DSw_NaCl_^−^ = *m_Cl_^−^/m*_0_ (3)

DSw = *m_w_/m*_0_ (4)

DSw_A_^−^ = *m_A_^−^*/*m_Cl_^−^* (5)

The released water percentage was calculated as the ratio of the gel weight after the anion exchange and equilibrated gel weight in NaCl, considering the mols of chlorine and anion balance. This was calculated from the MTAC content.

(6) $$W_{R}\left(\%\right)=\left(\frac{m_{A^{-}}}{m_{Cl^{-}}-\left(w_{Cl^{-}}+w_{A^{-}}\right)}\right)\times 100$$

where *w_Cl^−^_* and *w_A^−^_*, as the mass of anions, was calculated from the mols of anions, multiplied by its formula weight, according to *w* = *n* × *F_w_*.

The data for the anion exchange kinetic studies, followed by FTIR, were obtained from 3 independent experiments for reproducibility.

### 4.4. Methods

The Fourier transform infra-red (FTIR) spectra were obtained on a Frontier FTIR spectrometer, Perkin Elmer, and Nicolet 8600 in ATR mode using a ZnSe crystal. The wetting properties of the samples were characterized by a contact angle (CA) goniometer, OCA 35 (DataPhysics Ins., Filderstadt, Germany), using a 3 μL DW droplet and evaluated using the software, SCA202. All measurements were conducted in triplicate to ensure assay reproducibility. The DSC studies were performed on a Perkin–Elmer apparatus, DSC 8500. The ion diffused gel material samples were subjected to mechanical strain, and the break behavior was studied using a dynamic mechanical analyzer (DMA, TA-RSA-G2, TA Instrument) in the compression mode and axial direction. Equally sized test samples were cut, and the sample cylinders were placed on the stage of the DMA for analysis, in such a way that both the flat surfaces lay in the horizontal direction, while the tube-like cylindrical surface was settled in the vertical direction. This positioning ensured a homogenous distribution of the applied strain throughout the gel material. The test was conducted in triplicate to ensure reproducibility. Confocal Raman microscopy analyses were performed at a controlled room temperature of 23 °C using a WITec alpha 300 R+ confocal microscope, equipped with WITec UHTS300 spectrometers (WITec, Ulm, Germany), and the Carl Zeiss 50× objective was used. The focus layer was 100 µm below the surface layer of 1 mm thick gel samples. The 532 nm laser, with a power of 32 mW, was used for the excitation of the sample. The pyrene fluorescence was measured on a Shimadzu RF-5301PC spectrofluorophotometer, using 344 nm of excitation light. The prepared G1-3-Cl and G1-3-PFO gels were immersed in a 1 × 10^−5^ mol L^−1^ Py/water/MeOH solution (water/MeOH, 1/1 *v/v*) for several hours, until equilibrium in the Py concentration was reached in the gel and solution. The measurements were performed in the front-face arrangement in all cases. The XPS analysis was performed on a Thermo Scientific K-Alpha XPS system (Thermo Fisher Scientific, Altrincham, UK), equipped with a micro-focused, monochromatic Al Kα X-ray source (1486.6 eV). All other swelling experiments were performed by the immersion of the gel slabs in alcohol solvents in closed glass vials at room temperature. The experiments were conducted in triplicate to ensure reproducibility.

## Figures and Tables

**Table 1:** Hydrogel composition, swelling, and mechanical properties.

Sample ^1^	[MTAC] mol L^−1^	[HEAA] mol L^−1^	Crosslink. mol%	DSw_NaCl_^2^	DSw ^3^	σ at Break kPa	ε at Break %
G1-1-Cl	2	-	1.0	10.9	18.5	109.0 ± 10.6	40.0 ± 5.6
G1-2-Cl	2	-	1.5	5.9	13.1	424.0 ± 30.9	20.5 ± 3.8
G1-3-Cl	2	-	3.0	4.0	9.0	429.1 ± 28.6	13.5 ± 2.6
G1-5-Cl	2	-	4.5	3.0	6.3	485.0 ± 50.6	11.3 ± 2.2
G2-1-Cl	1	1	1.0	6.7	11.0	197.8 ± 16.6	52.9 ± 6.6
G2-2-Cl	1	1	2.0	4.0	7.9	420.5 ± 50.9	41.3 ± 3.9
G2-3-Cl	1	1	3.0	2.9	5.2	426.1 ± 28.1	28.0 ± 3.6
G2-5-Cl	1	1	4.5	2.1	4.9	432.7 ± 33.9	15.2 ± 4.2

^1^ The initiation of polymerization was performed using 1 mg of DMPA photoinitiator in all samples; ^2^ The degree of swelling in 0.9 wt% NaCl was calculated according to Equation (3); ^3^ The degree of swelling in deionized water was calculated according to Equation (4).

**Table 2:** Swelling properties of G1 and G2 hydrogels after the anion exchange.

Sample Code/ Counter-Anion	CaS		DS		PFO	
	DSw_A_^1^	W_R_^2^ %	DSw_A_^1^	W_R_^2^ %	DSw_A_^1^	W_R_^2^ %
G1-2	NA	NA	0.26	94.3	0.30	90.4
G1-3	1.1	−9.3	0.45	82.5	0.48	81.5
G2-1	1.1	−11.2	0.40	70.9	0.22	95.2
G2-2	NA	NA	0.32	91.1	0.32	92.1

^1^ The degree of swelling after the anion exchange (DSw_A_), calculated according to Equation (5); ^2^ The water loses after the anion exchange, calculated according to Equation (6).

**Table 3:** Water state characterization in the hydrogels, before and after the anion exchange.

Sample Code	Counterion Cl			CaS			DS			PFO		
	EWC ^1^	W_tf_^2^ %	W_nf_^3^%	EWC ^1^	W_tf_^2^ %	W_nf_^3^ %	EWC ^1^	W_tf_^2^ %	W_nf_^3^ %	EWC ^1^	W_tf_^2^ %	W_nf_^3^ %
G1-1	96.4	74.7	21.7	-	-	-			-	-	-	-
G1-2	94.0	73.6	20.4	-	-	-	31.7	7.3	24.5	34.8	14.7	20.1
G1-3	88.2	64.1	24.0	88.2	64.9	23.2	30.3	12.2	18.1	32.5	12.4	20.1
G1-5	83.8	59.0	24.8	-	-	-	-	-	-	-	-	-
G2-1	95.5	77.0	18.5	97.0	73.6	23.4	73.3	59.7	13.6	45.5	31.8	13.6
G2-2	91.2	66.7	24.5	-	-	-	52.8	32.4	20.4	45.4	34.1	18.7
G2-3	87.9	66.6	21.3	-	-	-	-	-	-	-	-	-
G2-5	83.8	62.1	21.7	-	-	-	-	-	-	-	-	-

^1^ Based on the freeze-drying of the sample; ^2^ Based on the DSC analysis; ^3^ Calculated according to Equation (2).

## Supplementary Materials

The following are available online at https://www.mdpi.com/article/10.3390/gels7030109/s1: Figure S1: ATR-FTIR spectra of the G1 and G2 gel series, measured as xerogels after drying. Figure S2: Representative photographs of the as-prepared gel samples, after washing in NaCl and after the DS anion exchange. Figure S3: Kinetics of the DS anion exchange, followed by FTIR at the absorption band at 1243 cm^−1^. The spectra were normalized to the 1535 cm^−1^ absorption band related to the polymer backbone. Figure S4: Raman signal of the G1-3 and G1-3_PFO gels, focusing on a different layer in the gel sample from that presented in the main article. Figure 5. Figure S5: Swelling behavior of the G1-3 and G2-1 gels in various alcohols: (a) G1-3-Cl and G2-1-Cl; and (b) G1-3-PFO and G2-1-PFO. The dashed line represents no weight or dimension change with respect to the equilibrated gels in a 0.9 wt% aq. NaCl solution. Video S1: Insertion of the G1-3-PFO hydrogel into the water surface and attempts at immersion. Figure S6: DSC thermograms of the G1-x-Cl and G2-x-Cl original gels and G1-3-CaS, G1-3-DS, G1-3-PFO, G2-1-CaS, G2-1-DS, and G2-1-PFO gels after the anion exchange.
