# Supplementary material for: Exchange Counterion in Polycationic Hydrogels: Tunability of Hydrophobicity, Water State, and Floating Capability for a Floating pH Device
Source: Gels. 2021 Aug 5;7(3):109. doi: 10.3390/gels7030109 (PMC8395738; doi:10.3390/gels7030109)
Supplement: Supplementary file 1 [file gels-07-00109-s001.zip › gels-1290513_supporting info.pdf]

Supporting information

Exchange counterion in polycationic hydrogels: tunability of hydrophobicity, water state, and floating capability toward for a floating pH device.

Martin Danko*[,1,2], Zuzana Kronekova[2], Igor Krupa[1], Jan Tkac[3], Peter Matúš[4], and Peter Kasak *[,1]

[1] *Center for Advanced Materials, Qatar University, P.O. Box 2713 Doha, Qatar; igor.krupa@qu.edu.qa (I.K.); peter.kasak@qu.edu.qa (P.K.)*

[2] *Polymer Institute, Slovak Academy of Sciences, Dúbravská cesta 9, 845 41 Bratislava, Slovak Republic; Martin.danko@savba.sk*

[3] *Institute of Chemistry, Slovak Academy of Sciences, Dúbravská cesta 9, 845 38 Bratislava, Slovak Republic; jan.tkac@savba.sk*

[4] *Institute of Laboratory Research on Geomaterials, Faculty of Natural Sciences, Comenius University in Bra-tislava, Mlynská dolina, Ilkovičova 6, 842 15 Bratislava, Slovak Republic; peter.matus@uniba.sk (P.M.).*

[*] *Correspondence: peter.kasak@qu.edu.qa; Tel.: 00974 44035674, P.K.; martin.danko@savba.sk,; M.D.*

Content:

Figure S1: ATR-FTIR spectra of G1 and G2 gel series measured as xerogel after drying.

Figure S2: Representative photographs of gel samples as prepared, after washing in NaCl and after DS anion exchange.

Figure S3: Kinetic of DS anion exchange followed by FTIR at absorption band at 1243 cm^{-1}. The spectra were normalized at 1535 cm^{-1} absorption band related to polymer backbone.

Figure S4: Raman signal of G1-3-Cl and G1-3-PFO gels focused in different layer in the gel sample as that presented in main article Figure 5.

Figure S5: Swelling behavior of G1-3 and G2-1 in various alcohols - (a) G1-3-Cl, G2-1-Cl and (b) G1-3-PFO, G2-1-PFO gels. Dashed line represents no weight or dimension change with respect to equilibrated gels in 0.9 wt% NaCl/water solution.

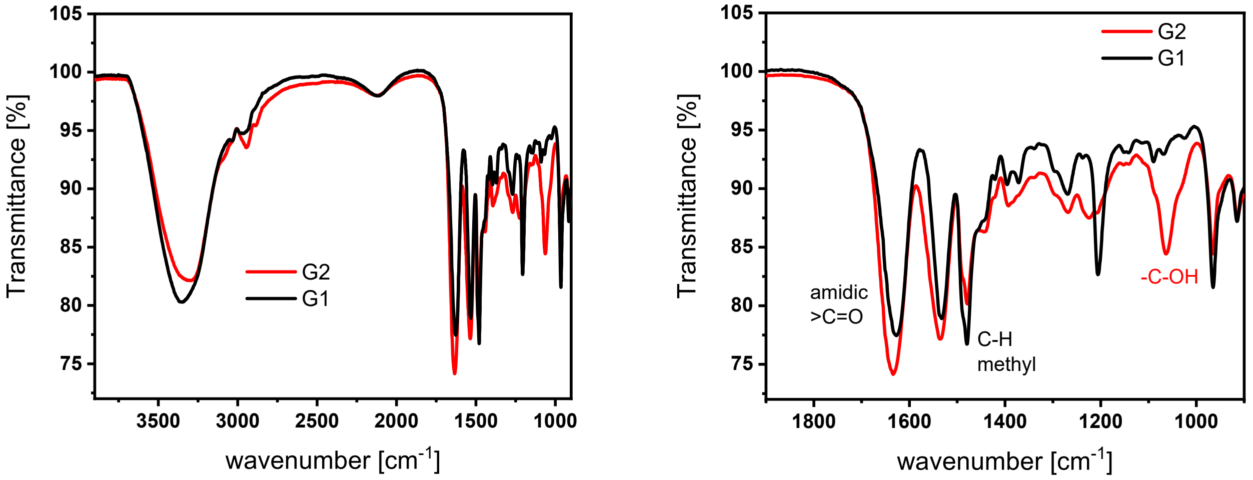

Figure S1: ATR-FTIR spectra of G1 and G2 gel series measured as xerogel after drying.

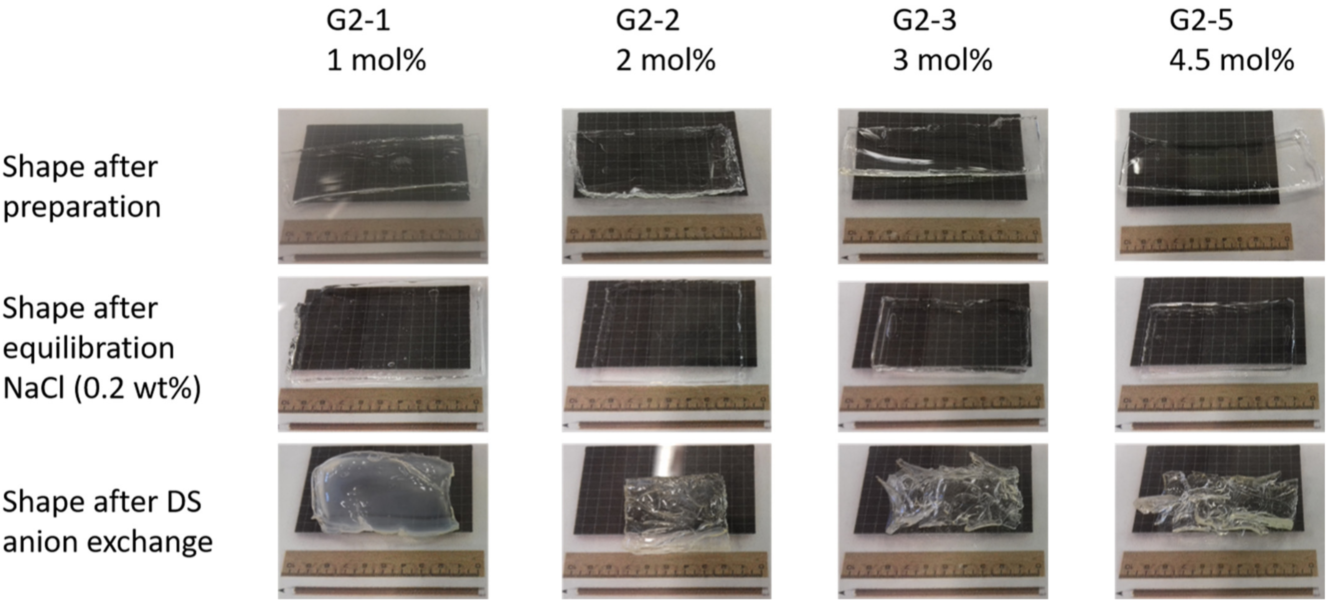

Figure S2: Representative photographs of gel samples as prepared, after washing in NaCl and after DS anion exchange.

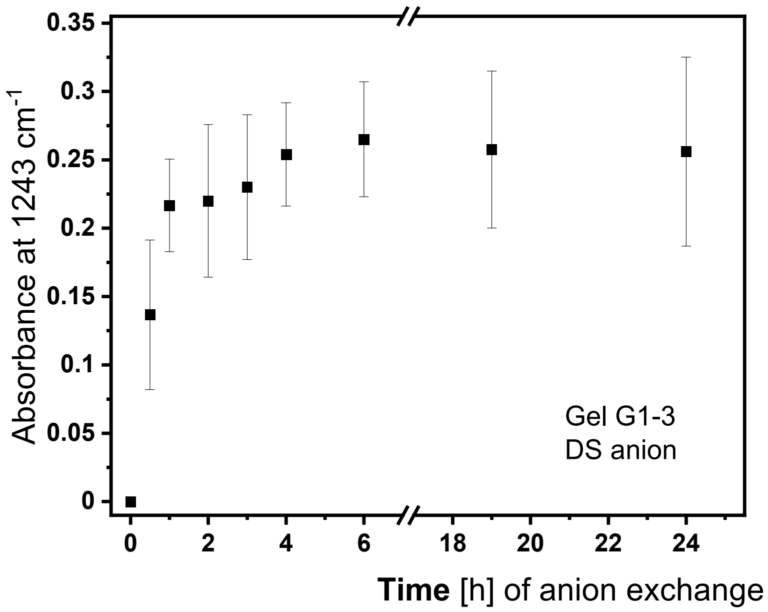

Figure S3: Kinetic of DS anion exchange followed by FTIR at absorption band at 1243 cm^{-1}. The spectra were normalized at 1535 cm^{-1} absorption band related to polymer backbone.

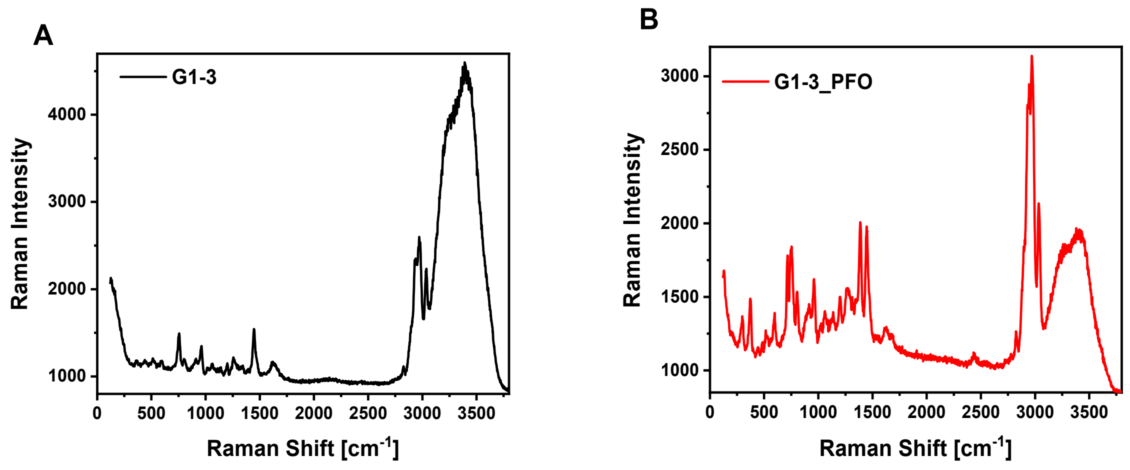

Figure S4: Raman signal of G1-3-Cl (A) and G1-3-PFO (B) gels focused in different layer in the gel sample as that presented in main article Figure 5.

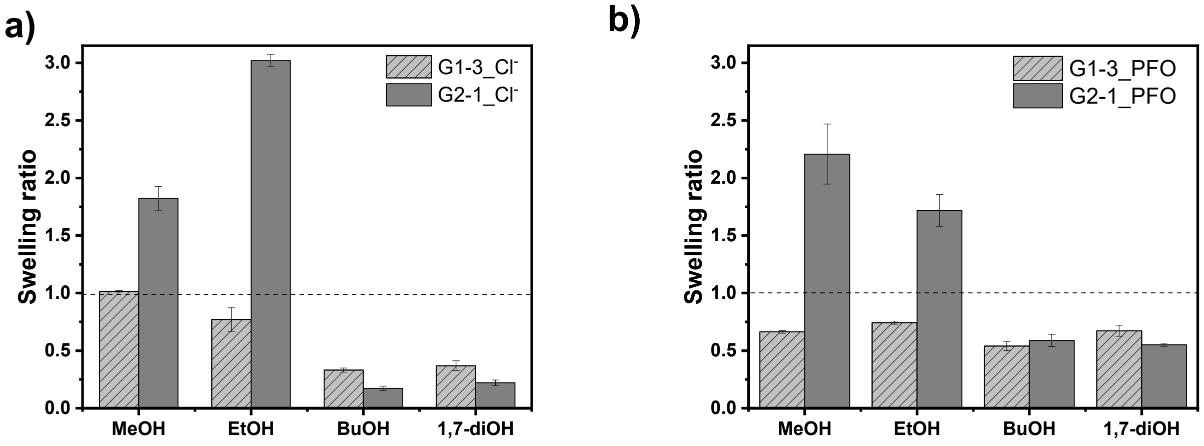

Figure S5: Swelling behavior of G1-3 and G2-1 in various alcohols - (a) G1-3-Cl, G2-1-Cl and (b) G1-3-PFO, G2-1-PFO gels. Dashed line represents no weight or dimension change with respect to equilibrated gels in 0.9 wt% NaCl/water solution.

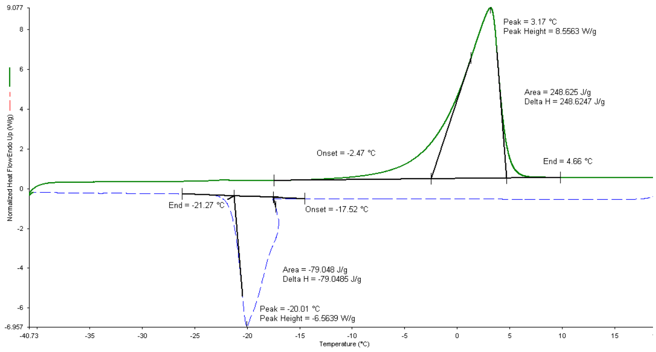

G1-1-Cl

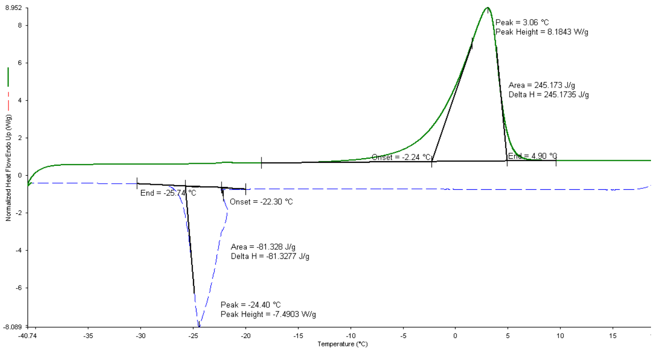

G1-2-Cl

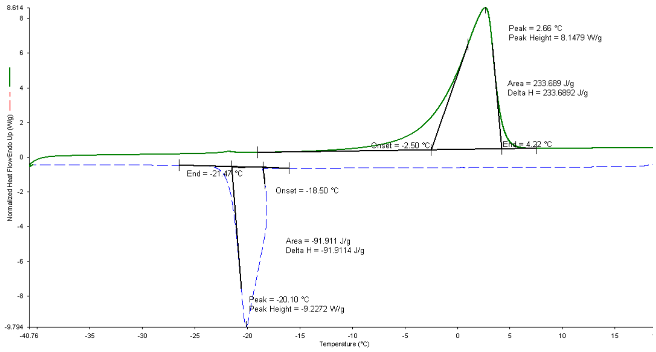

G1-3-Cl

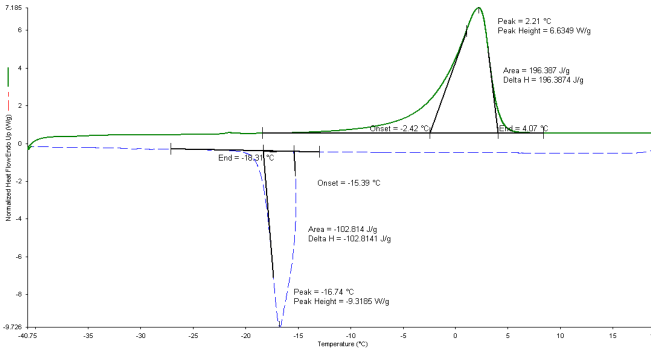

G1-5-Cl

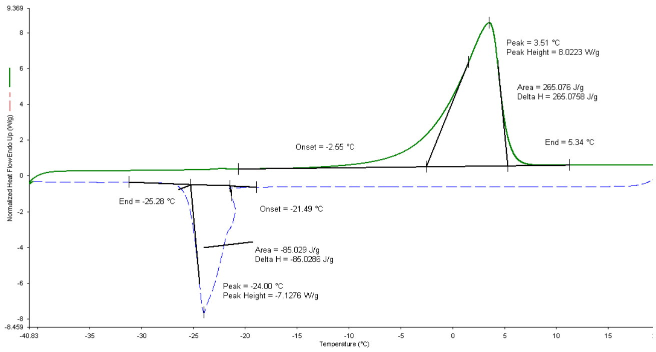

G2-1-Cl

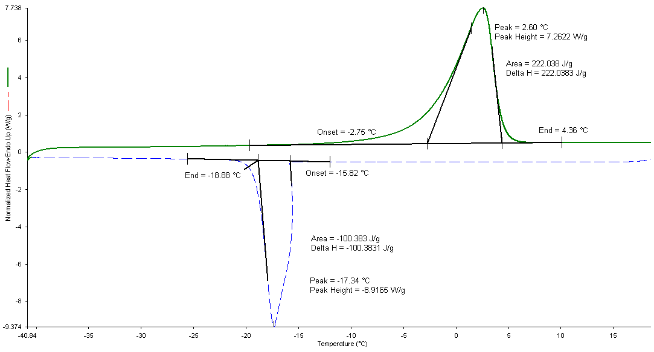

G2-2-Cl

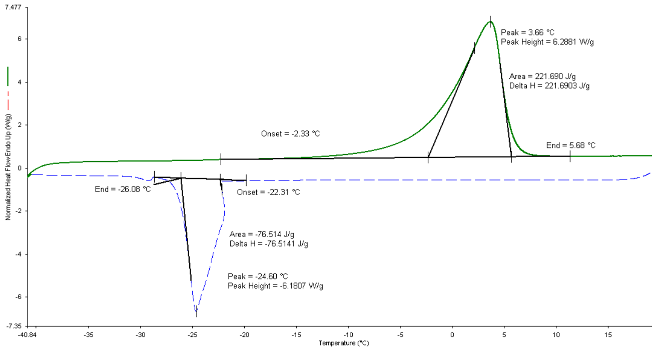

G2-3-Cl

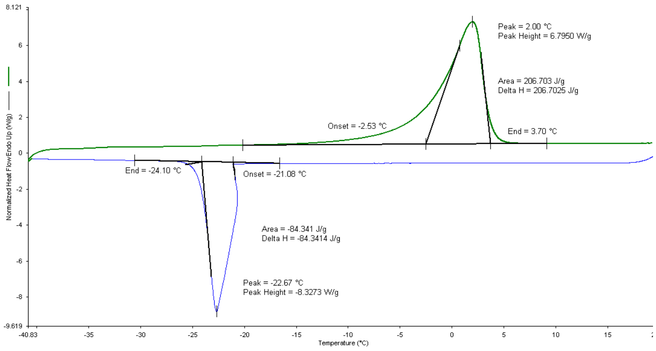

G2-5-Cl

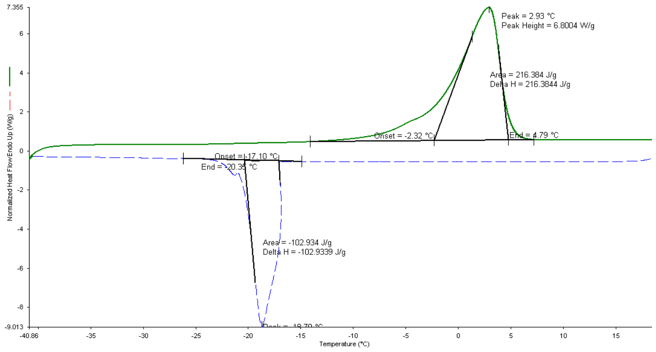

G1-3-CaS

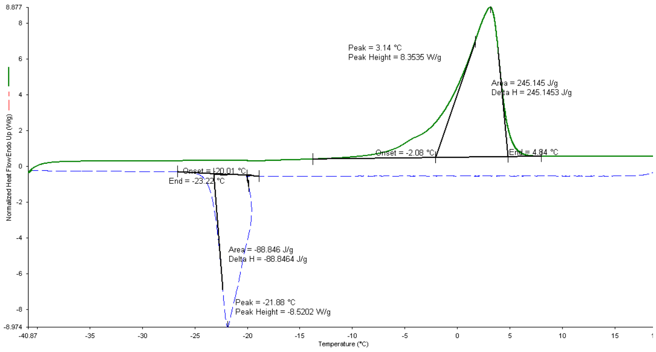

G2-1-CaS

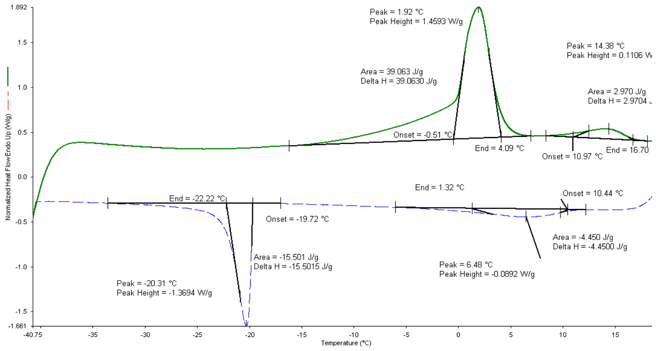

G1-3-DS

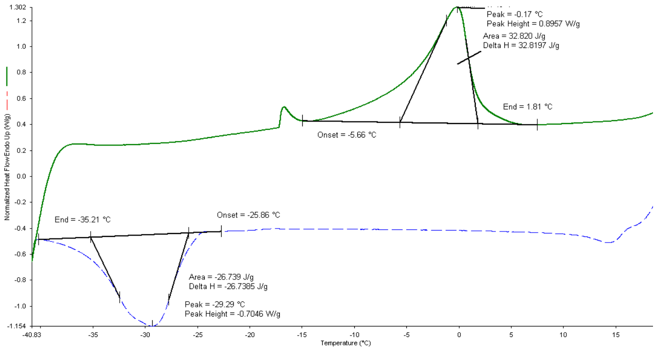

G2-1-DS

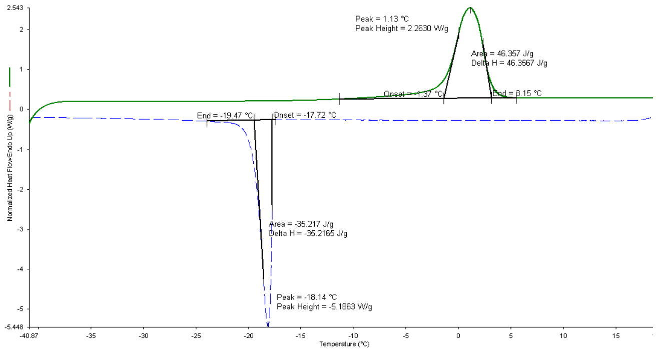

G1-3-PFO

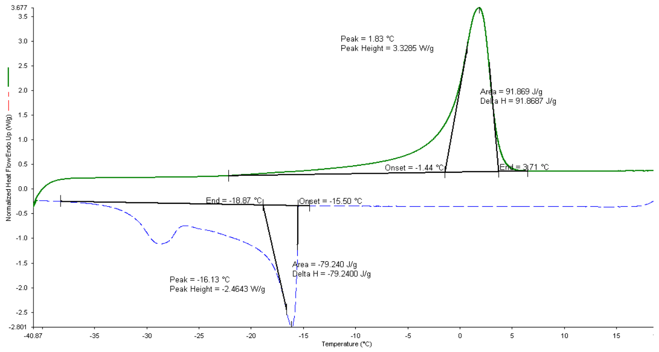

G2-1-PFO

Figure S6: DSC thermograms
